# Endoscopic injection vs anti-reflux surgery for moderate- and high-grade vesicoureteral reflux in children: a cost-effectiveness international study

**DOI:** 10.1007/s11701-024-02103-5

**Published:** 2024-10-16

**Authors:** F. Nascimben, F. Molinaro, M. Maffi, F. Nino, A. Lachkar, M. Zislin, M. Ogunleye, F. Becmeur, M. Messina, G. Cobellis, M. Lima, R. Angotti, I. Talon

**Affiliations:** 1 Service de Chirurgie Pédiatrique, Hôpitaux Universitaires de Strasbourg, Strasbourg, France; 2 Division of Pediatric Surgery, Department of Medical, Surgical and Neurological Sciences, University of Siena, Viale Bracci 14, 53100 Siena, Italy; 3 Pediatric Surgery Department, Istituti Di Ricovero E Cura a Carattere Scientifico (IRCCS) Azienda Ospedaliero-Universitraia Di Bologna, Bologna, Italy; 4 Pediatric Surgery, Salesi Children Hospital, Università Politecnica Delle Marche, Ancona, Italy

**Keywords:** Vesicoureteral reflux, Endoscopic injection, Ureteral reimplantation, Success rate, Costs, Radiation exposure

## Abstract

Even if vesicoureteral reflux is a common condition in children, there are no guidelines about the best therapeutic approach. This study aims to compare the results of endoscopic injection and ureteral reimplantation in children with grade III, IV and V VUR. A multicenter retrospective study included children with grade III, IV and V VUR treated from 2003 to 2018 at three Departments of Pediatric Surgery. Patients were divided into Group A (endoscopic injections) and Group B (anti-reflux surgery), B1 (open, OUR), B2 (laparoscopic, LUR) and B3 (robot-assisted laparoscopic RALUR). Follow-up was at least 5 years. 400 patients were included, 232 (58%) in group A and 168 (42%) in group B. Mean age at surgery was 38.6 months [3.1–218.7]. Mean follow-up was 177.8 months [60–240]. Group A had shorter operative time than group B (*P* < 0.01); lower analgesic requirement (*p* < 0.05), shorter hospital stay (*P* < 0.05) and lower overall costs (*p* < 0.05), but higher postoperative PNPs (*p* < 0.01), lower success rate (*p* < 0.01) and higher redo-surgery percentage (*p* < 0.01). No differences in terms of postoperative complications, success rate and mean radiation exposure between the two groups. Endoscopy is associated with shorter operative time, shorter hospitalization and lower cost, also in case of multiple injections. Recurrence rate after surgery is lower meaning lower rate of re-hospitalization and radiation exposure for children.

## Introduction

Vesicoureteral reflux (VUR) is an abnormal movement of urine from the bladder to the upper urinary tract, common in children [1] with an incidence of about 1–3% which may increase up to 15% in girls and 30% in boys in case of recurrent urinary tract infections (UTIs) [2]. VUR needs to be studied and staged to identify the best therapeutic option and prevent pyelonephritis and potential resulting reflux-linked nephropathies which may lead to chronic renal failure [3]. Different therapeutic options have been proposed: continuous antibiotic prophylaxis (CAP), endoscopic injection (EI) of bulking agents and anti-reflux surgery which can be open, laparoscopic or robotic [4]. Low-grade (I and II) VUR is generally treated with endoscopic injection as first line treatment [5], while high-grade (symptomatic IV and V) VUR with ureteral reimplantation (UR) [6], but there are no guidelines about the best approach for intermediate- and moderate-grade (III and IV) VUR. There is no definitive consensus for the best surgical approach to VUR in pediatric population.

## Aim of the study

This study aims to compare the outcomes of endoscopic injections (EI) and open (OUR), laparoscopic (LUR) or robot-assisted ureteral reimplantation (RALUR) in children with grade III, IV and V VUR through the analysis of a multicentric experience. It focuses the attention not only on the complications and the functional success rate, but also on the related costs and X-ray exposition of these two different approaches to identify the best treatment.

## Materials and methods

### Study population

In this multicenter international retrospective study, all pediatric patients younger than 18 years affected by primary moderate- and high-grade VUR (III, IV and V grade) treated between January 2003 and July 2018 at Pediatric Surgery Department of Strasbourg (FR), Bologna (IT) and Ancona (IT) were included.

Patients with low-grade VUR (I–II), secondary VUR due to solitary kidney, ectopic ureter, ureterocele, posterior urethral valve (PUV), duplex system, neurogenic bladder, severe voiding or bladder dysfunction, bladder exstrophy, primary obstructive mega-urehter (POM), patients with a history of previous pelvic surgery, reimplantation or endoscopic injection and patients followed up less than 5 years were excluded from the study.

Included patients were then divided into two main groups: Group A included patients treated with endoscopic injection at the first center, while Group B included patients treated with ureteral reimplantation at the second and the third centers. Group B was then subdivided into three subgroups: in B1 there were patients treated with an open approach (OUR), in B2 patients treated with a laparoscopic approach (LUR) and in B3 those who were treated with a robot-assisted ureteral reimplantation (RALUR).

### Demographic data, clinical presentation and imaging

The case records of all patients were retrospectively analyzed and all the following data were evaluated: gender, median age at diagnosis, Reflux Grade, laterality, prenatal diagnosis associated urinary anomalies, comorbidities and clinical presentation at diagnosis (symptoms at onset, number of pyelonephritis or febrile UTIs).

### Pre-operative management

All patients included in the study received diagnosis of III, IV or V grade VUR according to the European Pediatric Urology Classification on voiding cystourethrogram (VCUG). Ultrasound (US) and uro-magnetic resonance imaging (uro-MRI) were used to define the anatomy of the urinary tract and to exclude other urological comorbidities.

Febrile UTI was defined before and after surgery as a positive urine examination with a single bacteria with more than 10^5^ cfu/mL and more than 10^4^ leukocytes/ml and C-reactive protein (CRP) above 50 mg/l associated with fever above 38 °C. In cases of febrile UTIs, the functional impact was analyzed on dymercapto-succinil acid (DMSA) renal scan.

### Surgical technique

For all PATIENTS, median age at surgery, surgical technique (endoscopic injection, open, laparoscopic or robotic reimplantation), type of bulking agent, number of endoscopic injections, intra and peri-operative complications were analyzed. The therapeutic approach was determined according to the center’s habits in treating grade III, IV and V vescicoureteral reflux.

- A. Endoscopic injection
  Patients underwent endoscopic correction under general anesthesia as a single-day procedure. They were placed in the lithotomy position. Surgeons used a 9.5-Fr pediatric cystoscope and through a 3.7-Fr metallic needle hyaluronic acid and dextran copolymer (Deflux) was injected submucosally at the 6 o’clock position to create a bulge. In most patients, only 1 puncture at 6 o’clock was sufficient, but in rare cases of inadequate sub-ureteral, another puncture was performed at a different location. In cases of duplication, injection was done under the refluxing ureter. The mean amount of each substance injected into the ureter was defined according to reflux grade or shape of the ureteral orifice. An intra-operative control VUCG was performed to check eventual residual reflux.
- B. Ureteral reimplantation
  Ureteral reimplantation was performed under general anesthesia too with the patient placed supine.

- The open submucosal reimplantation was performed according to Cohen technique and it consisted of an open bladder procedure with a submucosal channel designed to be at least three times the diameter of the ureter [7].
- The laparoscopic approach consisted in an extra-vesical reimplantation according to Lich Gregoir with three 3–5 mm laparoscopic ports. The camera port was inserted at the umbilicus and then CO_2_ pneumo-peritoneum was created for the insertion of the other ports under laparoscopic view. Five millimeter 30° laparoscope and standard 3 mm laparoscopic instruments were used for the reimplantation procedure. An incision was made in the peritoneum just above the posterior bladder wall on the affected ureter. The ureter underlying on a loop was mobilized by careful dissection to avoid injuring the deferens vas or the uterine artery. After the isolation of the ureter, the surgeon prepared a mucosal tunnel in the bladder wall. The bladder was distended with approximately 50 ml of sterile physiological saline through the bladder catheter. The detrusor muscle was then incised using electrocautery in a layered fashion, thereby preserving the bladder mucosa. Once the mucosa was exposed, the surgeon re-approached the detrusor muscle over the ureter and performed a ureteral anastomosis with separated stitches Vicryl 3/0. Before completing anastomosis, a double J stent was placed into the ureter. Detrusor muscle was then re-approximated over the ureter. In bilateral malformations, ureters were reimplanted in the same way in a common mucosal tunnel [8].
- The same procedure was performed through the robot-assisted approach. Using open access, four laparoscopic ports were placed; the first 12 mm trocar for camera port was placed at the umbilicus with one 8 mm working trocar placed on the right flank 1 cm above the umbilical line along the mid-clavicular line and the other in the contralateral position. Da Vinci robot was docked over the patient’s feet.

### Post-operative outcome and long-term follow-up

Primary outcomes included post-operative complications occurring during the hospitalization and/or re-hospitalization within 90 days, post-operative pain control, use of antibiotics, time of vescical catheter stay (VCS) and time of drainage stay (DS), and length of hospital stay (LOS).

Secondary outcomes included recurrent VUR, re-do surgery, costs and radiation exposure.

All patients underwent ultrasonography and clinical examination 1, 6 and 12 months after discharge. In case of EI, last post-operative control was done 5 years after surgery.

1. Success rate
  Successful reflux correction was defined as absent reflux or lower reflux on follow-up. At center A, in case of primary injection failure, because of recurrent UTIs or persistent VUR, a second injection was performed, and those children for whom a second injection failed underwent a third injection. Surgery was performed in case of local causes, such as diverticulum or uretherocel, after vescico-ureteral junction iatrogenic obstruction or after 3 unsuccessful EIs. At center B, in case of failure of ureteral reimplantation, a new reimplantation or an uretero-nephrectomy was performed.
2. Costs
  The cost of the single procedure (endoscopic injection or ureteral reimplantation), the cost of the total hospital stay linked to the different surgical procedures and the cost for single patients considering the recurrence rate were analyzed. Total direct cost was calculated by summing the cost of all individual billing items provided in the charge master for each procedure. Costs were tabulated for the 15 years following both endoscopic injection and ureteral reimplantation. These costs were further subdivided into surgery and operating room use, imaging exams, laboratory, postoperative complications requiring re-admission to the Hospital.
3. Radiation exposure
  All patients underwent a first VUCG to confirm the suspected diagnosis of VUR and define the VUR grade before surgery. In case of endoscopic injection, one or more X-rays with medium contrast were performed immediately after each injection in the operating room to detect the persistence of VUR or the bulking agent-urinary obstruction (center A). In case of recurrence of febrile UTI after surgery, VUCG was performed to evaluate the presence of residual VUR and check if another procedure was necessary.

### Statistical analysis

Patients’ characteristics were presented using mean, percentage and range for continuous variables and frequencies for categorical variables. The comparison of the groups was assessed using the Fisher test for categorical variables and *t* test for continuous variables. For all tests *p* value < 0.05 was considered as significant.

## Results

### Study population

A total of 400 children, (626 treated ureters) with primary grade III, IV and V VUR and surgically treated between 2003 and 2018 at the Department of Pediatric Surgery in Strasbourg (France), Bologna (Italy) and Ancona (Italy) were included in the study. 179 (44.7%) were girls and 221 (55.3%) boys. Median age at surgery was 38.63 months [2.13–218.7 months]. 226 (56.5%) patients had bilateral VUR and 174 (43.5%) unilateral VUR, VUR was on the left side in 112 children (64.37%) and on the right side in the other 62 cases (35.63%). 84 patients (21%) received prenatal diagnosis of urinary anomalies (hydronephrosis) and 11 (2.75%) had familiarity for urological pathologies. There were 225 (56.25%) cases of grade III VUR, 136 (34%) cases of IV grade VUR and 39 (9.75%) cases of grade V. 75 (18.75%) patients were completely asymptomatic and the other 325 (81.25%) had at least one episode of febrile UTIs or PNF, specifically 102 (25.5%) had only one episode of PNF, 133 (33.25%) had 2 episodes of PNF, 57 (14.25%) had 3 and 34 (8.5%) had more than 3 episodes. Mean length of follow-up was 177.8 months (from 60 to 240 months). Table 1 reports all data about the whole population included in the study.

**Table 1:** Demographic data for all included patients

Parameter	*N* (%)
Tot	400
*Sex*	
M	221 (55.3)
F	179 (44.7)
Age, median, months (range)	38.6 [2.1– 218.7]
*Familiarity*	
Yes	11 (2.75)
No	389 (97.25)
*Prenatal diagnosis*	
Yes	84 (21)
No	316 (79)
*Laterality*	
Unilateral	174 (43.5)
Left	112 (28)
Right	62 (15.5)
Bilateral	226 (56.5)
*VUR grade*	
III	225 (56.25)
IV	136 (34)
V	39 (9.75)
*PNF or febrile UTIs*	
0	75 (18.75)
1	102 (25.5)
2	133 (33.25)
3	57 (14.25)
> 3	34 (8.5)

### Comparison between groups

#### Demographic data and pre-operative management

232 (58%) patients underwent endoscopic injection (EI) at first center and were included in the group A, 168 (42%) patients were surgically treated with ureteral reimplantation (UR) at the other two centers and they were included in group B. Specifically 92 children (54.76%) underwent open procedure (OUR) according to Cohen technique, 41 (24.40%) the laparoscopic ureteral reimplantation (LUR) according to Lich–Gregoir technique and 35 (8.75%) the robot-assisted laparoscopic ureteral reimplantation (RALUR).

There are no differences between Group A and Group B in terms of age at surgery, gender, prenatal diagnosis, familiarity, comorbidities or associated urological anomalies (Table 2). Results comparing group A with group B1, B2 and B3 are reported in Table 3. On the other hand, VUR grading is significantly higher in the UR group in which over 60% of the cases had grade IV or V VUR, while in the EI group, only 30% had VUR grading higher than III (*P* < 0.0001). This difference is more evident if we compare the VUR grading of group A (grade III in 68.1% of cases) with that of group B2 (grade IV in 68.3%) and B3 (grade IV in 45.7% and V in 25.7%). Furthermore, there are significantly more symptomatic patients in the EI group than in UR one (94.4% vs 63.1%; *p* = 0.00001). The incidence of hydronephrosis was significantly higher in the UR group (28.9% vs 12.5%, *P* = 0.00001). Significantly more renal scarrings were detected in the reimplantation than EI group (26.8% vs 33.3%, *P* = 0.001).

**Table 2:** Demographic data and pre-natal management, comparison between group A (endoscopic injection, EI) and group B (ureteral reimplantation)

	EI (Group A) n = 232	UR (Group B) n = 168	P value
Sex, male (*n*, %)	121 (52.2)	100 (59.5)	*p* = 0.1
Mean age (months)	38.1 [1–173.5]	39.2 [2.1–218.7]	*p* = 1
Familiarity (*n*, %)	5 (2.2)	6 (3.6)	*P* = 0.3
Prenatal diagnosis (*n*, %)	53 (22.8)	31 (18.5)	*P* = 0.3
Laterality, bi (*n*, %)	110 (47.4)	95 (56.5)	*P* = 0.07
*VUR grade* (*n*, %)			
III	158 (68.1)	67 (39.9)	*P* < 0.001
IV	64 (27.6)	72 (42.9)	*P* = 0.001
V	10 (4.3)	29 (17.3)	*P* < 0.01
Urinary anomalies (*n*, %)	54 (23.3)	34 (20.2)	*P* = 0.4
Comorbidities (*n*, %)	16 (6.9)	12 (7.1)	*P* = 0.9
PNFs or febrile UTIs (*n*, %)	219 (94.4)	106 (63.1)	*P* < 0.01
US hydronephrosis	67 (28.9)	13 (12.5)	*P* < 0.01
DMSA renal scars	44 (18.9)	56 (33.3)	*P* = 0.001

**Table 3:** Demographic data, comparison between group A (endoscopic injection) and group B 1 (open ureteral reimplantation), B 2 (VLS ureteral reimplantation), B 3 (robot-assisted ureteral reimplantation)

	EI (Group A) *n* = 232	OUR (Group B1) *n* = 92	LUR (Group B2) *n* = 41	RAUR (Group B3) *n* = 35	*P* value
Sex, male (*n*, %)	121 (52.2)	53 (57.6)	23 (56.1)	24 (68.6)	*P* = 0.3
Mean age (months)	38.1 [1–173.5]	35.6 [2.1–218.7]	39.9 [5.2–151.5]	48.6 [6.5–147.9]	
Familiarity (*n*, %)	5 (2.2)	4 (4.3)	1 (2.4)	1 (2.9)	*P* = 0.7
Prenatal diagnosis (*n*, %)	53 (22.8)	16 (17.4)	9 (21.9)	6 (17.1)	*P* = 0.6
Laterality, bi (*n*, %)	110 (47.41)	53 (57.6)	20 (48.8)	22 (62.9)	
*VUR grade* (*n*, %)					
III	158 (68.1)	46 (50)	10 (24.4)	10 (28.6)	*P* < 0.001
IV	64 (27.6)	30 (32.6)	28 (68.3)	16 (45.7)	*P* < 0.001
V	10 (4.3)	16 (17.4)	3 (7.31)	9 (25.7)	*P* = 0.0001
Urinary anomalies (*n*, %)	54 (23.3)	20 (20.7)	8 (19.5)	6 (17.1)	*P* = 0.8
Comorbidities (*n*, %)	16 (6.9)	7 (7.6)	3 (7.3)	2 (5.7)	*P* = 1
PNFs or febrile UTIs (*n*, %)	219 (94.4)	58 (63)	20 (48.8)	29 (82.9)	*P* < 0.001

### Surgical data

Endoscopic injection was associated with a significantly shorter mean operative time than ureteral reimplantation (28.9 min vs 161.2 min, *P* < 0.00001), OUR (115 min), LUR (198.5 min) and RALUR (240 min). No intraoperative complications were detected in all groups. There were no conversions to open surgery in B2 and in B3 group. The percentage of boys who underwent concomitant circumcision in EI group and in UR group were 39.2% and 2.97%, respectively (*P* = 0.00001).

### Primary outcome

**Fig. 1:**
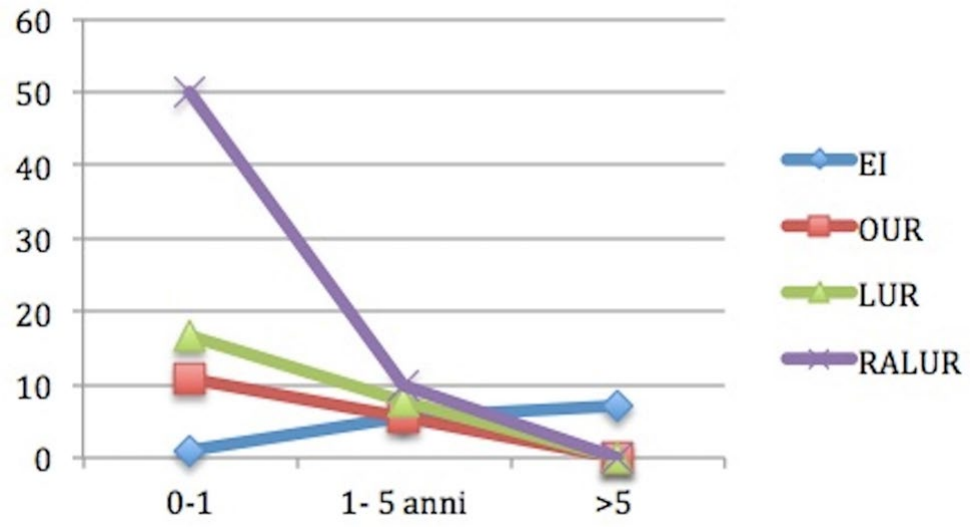
It shows the complication rate for each group based on age at surgery. We can notice that complication rate in EI group has the tendence to increase with the increasing age (1.4% < 5.6% < 7%), while the opposite tendence can be seen in group B1 (10.8% > 5.4% > 0%), B2 (16.7% > 8% > 0%) and B3 (50% > 10% > 0%). No complications are seen in patients older than 5 years treated with surgery (open, laparoscopic or robotic-assisted laparoscopic surgery)

The mean follow-up for the total patients was 177.8 months [60–240 months]. During this period, a total of 21 (5.25%) patients experienced complications; 10 of 232 (4.31%) patients in group A and 11 of 168 (9.52%) in group B. In group A complications involved 9 (3.87%) cases of meatal obstruction due to iatrogenic calcification managed by double J stent insertion in 5/8 cases and 1 (0.43%) cases of persistent hematuria. In group B, there were 5 cases (2.97%) of de novo hydroureteronephrosis, 2 (1.19%) cases of obstructive megauretere, 1 (0.59%) of persistent hematuria, 1 (0.59%) of hypertension, 1 (0.59%) of abdominal would dehiscence and 1 (0.59%) of accidental removal of JJ stent. There were no cases of conversion. There was no a statistically significant difference between the two groups (*P* = 0.16). The complications analyzed during the first 90-day follow-up in group A, B, B1, B2 and B3 are shown in Table 4. In Fig. 1, we can see the complication rate in all the groups stratified for age (0–1 year, 1–5 years and > 5 years); we can notice that the complication rate increases with the increasing of age at surgery for EI, but it decreases in B group (the tendency to decrease is higher in B3 than in B2 than in B1).

**Table 4:** 90-day post-operative complications, comparison between group A (endoscopic injection), B1 (open ureteral reimplantation), B2 (laparoscopic ureteral reimplantation) and B3 (robotic ureteral reimplantation)

	EI (Group A) *n* = 232	UR (Group B)	OUR (Group B1) *n* = 92	LUR (Group B2) *n* = 41	RAUR (Group B3) *n* = 35	*P* value
TOT, *n* (%)	10 (4.31)	11 (9.52)	4 (4.34)	4 (9.75)	3 (8.57)	*P* = 0.22
Obstruction, *n* (%)	9 (3.87)	0	0	0	0	*P* = 0.33
Persistent hematuria, *n* (%)	1 (0.43)	1 (0.59)	1 (1.08)	0	0	*P* = 0.99
Abdominal wound dehiscence, *n* (%)	0	1 (0.59)	1(1.08)	0	0	*P* = 0.81
Accidental removal of JJ stent, *n* (%)	0	1 (0.59)	0	0	1 (2.85)	*P* = 0.92
Hydroureteronephrosis, *n* (%)	0	5 (2.97)	1 (1.08)	3 (7.31)	1 (2.85)	*P* = 0.08
Hypertension, *n* (%)	0	1 (0.59)	0	0	1 (2.85)	*P* = 0.42
Obstructive megauretere, *n* (%)	0	2 (1.19)	1 (1.08)	1 (2.43)	0	*P* 0 O.81

For postoperative pain control, there was a significantly higher analgesic use in group B than in group A calculated as the total median days of analgesic requirement (1.8 days vs 4.7, *P* < 0.0001) and type of analgesics used (Paracetamol was sufficient to control post-operative pain in 98.8% of endoscopic injections vs 78.6% in ureteral reimplantation group, in fact 132/168 patients needed non-steroidal anti-inflammatory drug and morphine, *P* < 0.00001). Pain was better controlled after minimally invasive surgery (B2, B3) than after the traditional one (B1) (*P* < 0.0001). In group A, no drainage or vescical catheter was left in place at the end of the procedure, instead UR group reported the use of vescical catheter in 100% of cases for a mean time of 5.35 days [2–9] and drainage in 98.2% of cases for a mean time of 2.34 days [0–11]. Length of hospital Stay (LOS) was shorter in group A than in group B (median LOS was 1.03 [1–3] days vs 7.1 [5–14]; *p* < 0.0001). Data about primary outcome (LOS, analgesic requirement, drain stay, CV stay, use of antibiotic) for each group are reported in Table 5**.**

**Table 5:** Primary outcome, comparison between group A (endoscopic injection), B1 (open ureteral reimplantation), B2 (laparoscopic ureteral reimplantation) and B3 (robotic ureteral reimplantation)

	EI (Group A) *n* = 232	UR (Group B)	OUR (Group B1) *n* = 92	LUR (Group B2) *n* = 41	RAUR (Group B3) *n* = 35	*P* value
LOS (days)	1.03 [1–3]	7.1 [4–14]	8.14 [5–14]	6.81 [5–12]	7.07 [4–13]	*P* = 0.016
Analgesics (days)	1.8 [1–4]	4.7 [2–12]	6.67 [4–10]	4.92 [2–12]	3.86 [2–6]	*P* = 0.8
Vesical drain (days)	0	5.35 [1–9]	5.65 [2–9]	5.3 [1–9]	5.11 [3–7]	*P* = 0.07
Drainage (days)	0	2.34 [0–11]	3.14 [2–11]	2.04 [0–4]	2.34 [0–5]	*P* = 0.6
Antibiotics (days)	1.2 [1–3]	7.53 [4–20]	9.94 [5–20]	6.7 [4–11]	7.41 [6–15]	*P* = 0.4

### Secondary outcome and long-term follow-up

**Fig. 2:**
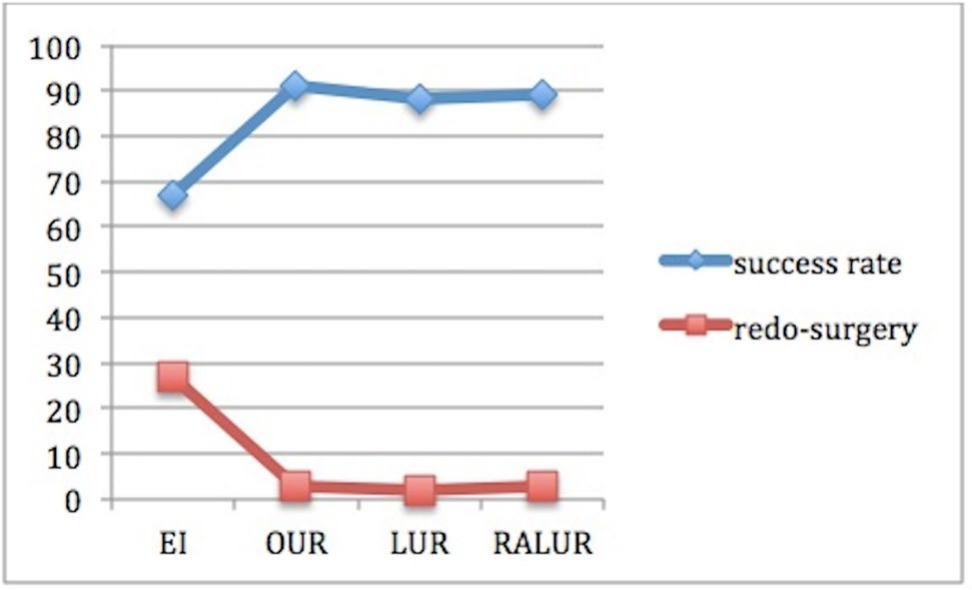
Success rate and incidence of redo-surgery after the first endoscopic injection (group A), open ureteral reimplantation (group B1), laparoscopic ureteral reimplantation (group B3) and robot-assisted laparoscopic ureteral reimplantation (group B3)
  If we analyze the role of PNPs, VUCG and renal scan in detecting VUR recurrences after EI and UR, we can see important differences. PNPs were recorded in 43.61% (*n* = 41) of cases, 46.74% after the first injection in group A and 29.41%) in group B (*p* = 0.013) with a statistically significant difference among B1, B2 and B3 group (12.5% vs 40% vs 50%, *p* < 0.01). Examining the role of postoperative VUCG in detecting persistent/recurrent VUR, there was not a significant difference between group A and group B (24.67% vs 35.29%; *p* = 0.12) and among group B1, B2 and B3 during follow-up (*P* = 0.68). De novo contralateral VURs were found in 11 patients (4.74%) after first EI and in 2 patients (1.19%) after ureteral reimplantation group respectively (*P* = 0.21). New renal scarring was found in 22 patients (28.57%) in group A and in 6 patients (35.29%) in group B (*p* = 0.45).
  The overall rate of redo-surgery (RRS) was 17% (*n* = 68/400), 27.15% in group A and 2.97% in group (*p* < 0.001); specifically 3.26% in B1, 2.41% in B2 and 2.85% in B3) (Fig. 2). RRS increased after each EI: 27.15% after the I EI, 36.5% after the II and 68.75% after the III one. The SR after the UR performed in 7 patients after 2 injections and in 11 after 3 was 100%.
  11.76% of failed cases in group B was treated with EI, while the other 29.41% with UR.
  Age at surgery plays an important role in determining SR and RRS: SR decreases with the increasing of age at surgery for EI, but it increases in in the other groups, RRS had the opposite trend: it increases with the increasing age in A group, but it decreases in B1, B2 and B3 (Figs. 3, 4).

1. Success rate
  The global success rate (SR) defined as VUR downgrading or VUR resolution and absence of symptoms was 76.5% (*n* = 306/400): 66.81% in group A vs 89.88% in group B (*p* < 0.01); 91.3% in B1 vs 87.8% n B2 vs 88.57% in B3 (*p* = 0.8) (Fig. 2).

**Fig. 3:**
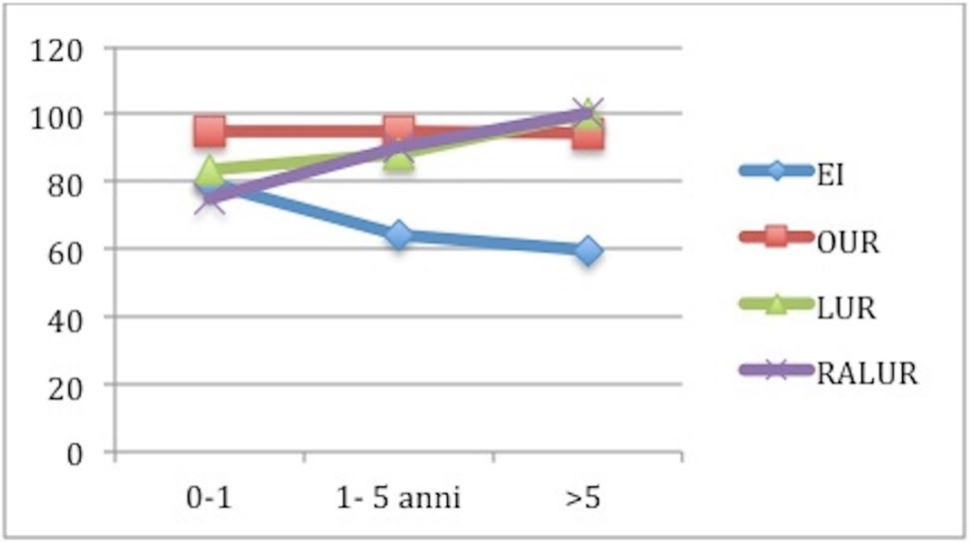
It shows the success rate for each group based on age at surgery. We can notice that success rate in EI group has the tendence to decrease with the increasing age (79.5% > 64% > 59.7%); in B1, it is the same at any age (94.6% = 94.6% = 94.4%), while the opposite tendence can be seen in group B2 (83.4% > 88% > 100%) and B3 (75% > 90% > 100%). Specifically, laparoscopic and robotic-assisted laparoscopic surgery succeed in 100% of cases in patients older than 5 years

**Fig. 4:**
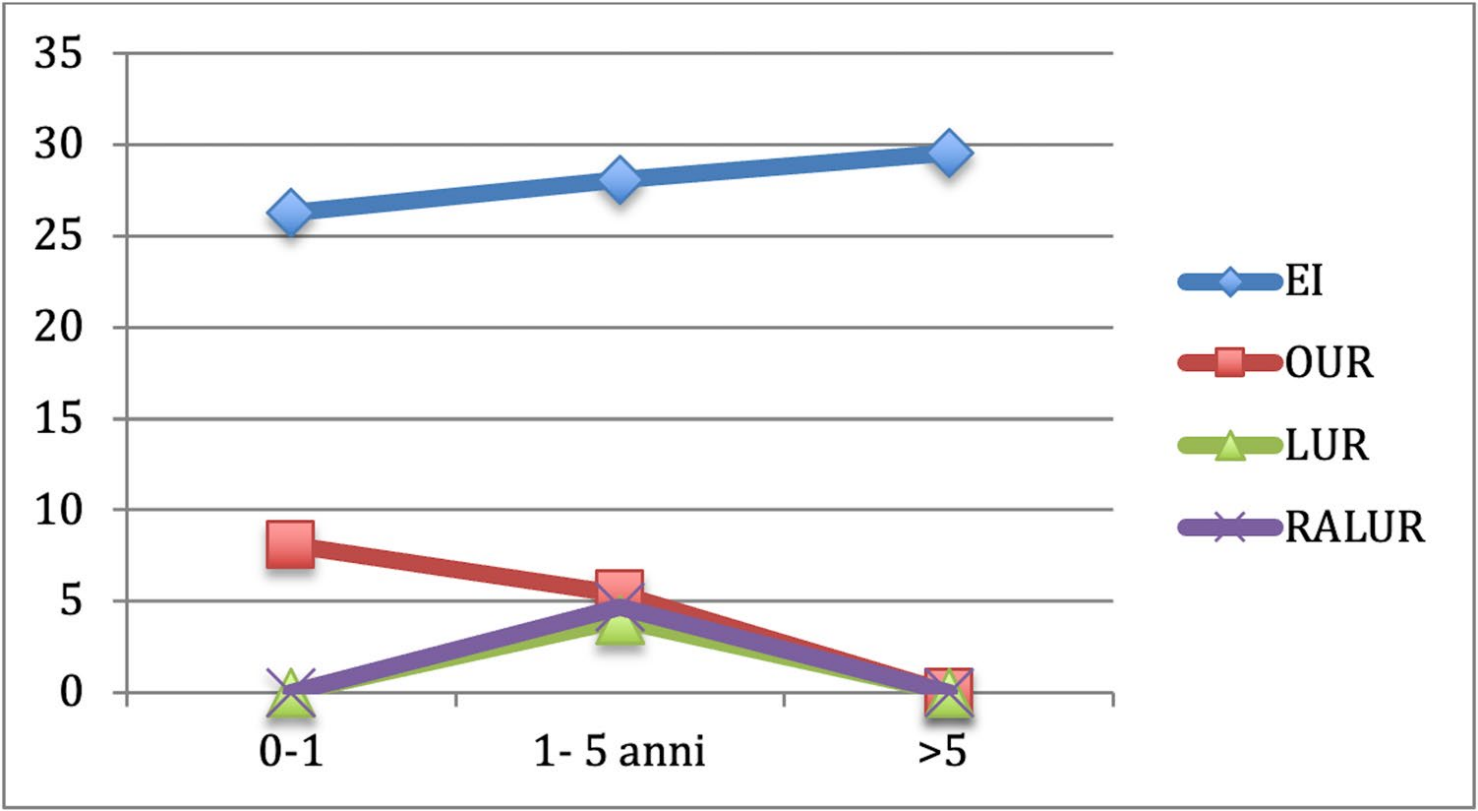
It shows the re-do surgery rate for each group based on age at surgery. We can notice that re-do surgery rate in EI group has the tendence to increase with the increasing age (26.3% < 28.1% < 29.6%), while the opposite tendence can be seen in group B1 (8.1% > 5.4% > 0%), B2 (0% > 4% > 0%) and B3 (0% > 4.7% > 0%). No re-do surgery is needed in patients older than 5 years treated with surgery (open, laparoscopic or robotic-assisted laparoscopic surgery)
  Further subgroup analyses across the different VUR gradings showed that VUR grade does not influence the SR and the RRS of B1, B2 and B3, but it does in A group; specifically in A group success rate decreases and re-do surgery rate increased with the increasing grade of VUR (Figs. 5, 6).

**Fig. 5:**
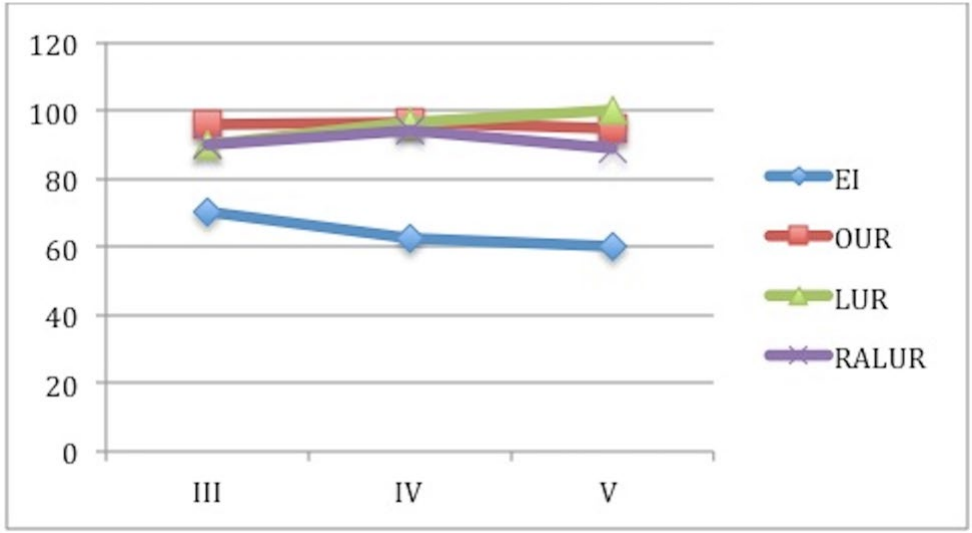
It shows the success rate for each group (A, B1, B2 and B3) based on VUR grade. We can notice that success rate in EI group decreases with the increasing VUR grade (70% > 62.5% > 60%), while it is very similar in the other groups if we consider the different grades of VUR (95.7% vs 96.4% vs 94.4%), B2 (90% vs 96.4% vs 100%) and B3 (90% vs 93.8% vs 88.9%)

**Fig. 6:**
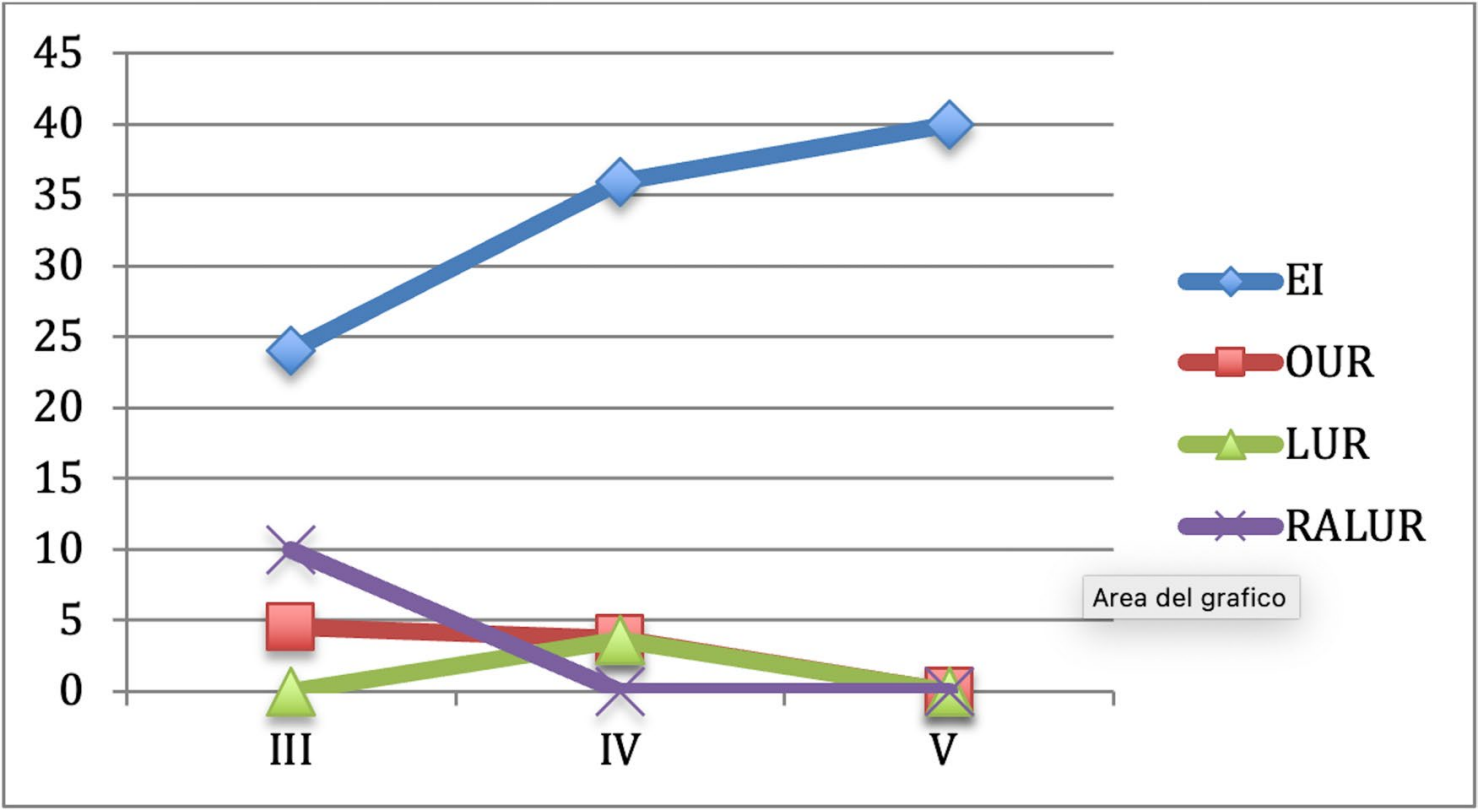
It shows the re-do surgery rate for each group (A, B1, B2 and B3) based on VUR grade. We can notice that re-do surgery rate in EI group increases with the increasing VUR grade (24% < 35.9% < 40%), while it is very similar in the other groups if we consider the different grades of VUR; B1 (4.3% vs 0% vs 0%), B2 (0% > 3.6% > 0%) and B3 (2.85% > 0% > 0%)
2. Costs
  The median cost of a single endoscopic injection was 659 euros, whereas the median cost of a single open ureteral reimplantation was 5000 euros, of a laparoscopic one 5200 euros and of a robotic one 5700. A multivariate analysis of cost found that open ureteral reimplantation increased total costs of EI by 86%, laparoscopic UR by 87% and robot-assisted UR by 88%. A subcomponent analysis of total cost revealed that the cost of the entire hospitalization including the room, the board costs, the therapeutic costs was 1331 for EI, 7300 for open surgery, 8600 for laparoscopic one and 9100 for the robotic one. Median total cost was higher in B group than in A group with a statistically significant difference (*p* < 0.01) and in B3 than in B2 and B1 even if without difference (*p* = 0.07).
  If we analyze the frequency of complications, patients with complications had a higher probability of longer hospital stay, longer use of analgesics and antibiotics and higher total costs. Even if the probability of any complication is higher in UR group, this difference is not significant and it does not interfere with our results.
  On the other hand, if we consider additional procedures, related exams and hospitalizations, median total cost was still substantially higher in group B than in group A. When two or more injections were done, the final cost for each patient does not significantly increase. Among 232 patients in group A, one injection was sufficient for 169, while 63 needed two injection and 16 three with a median of 1.47 injection for patient for a median total cost of 1956 euros which is still lower than the median total cost of UR (8999 euros) with a median of 1.08 procedure for patient. Specifically the median total cost for an open UR was 7519 euros with a median of 1.03 procedures for patient, for a laparoscopic Ligh Gregoir was 8772 euros with a median of 1.02 procedure for patient and for a robotic UR was 9373 euros with a median of 1.03 procedure for patient without any significant difference (*p* > 0.05).
  Total and partial median costs for each group are shown in Fig. 7.

**Fig. 7:**
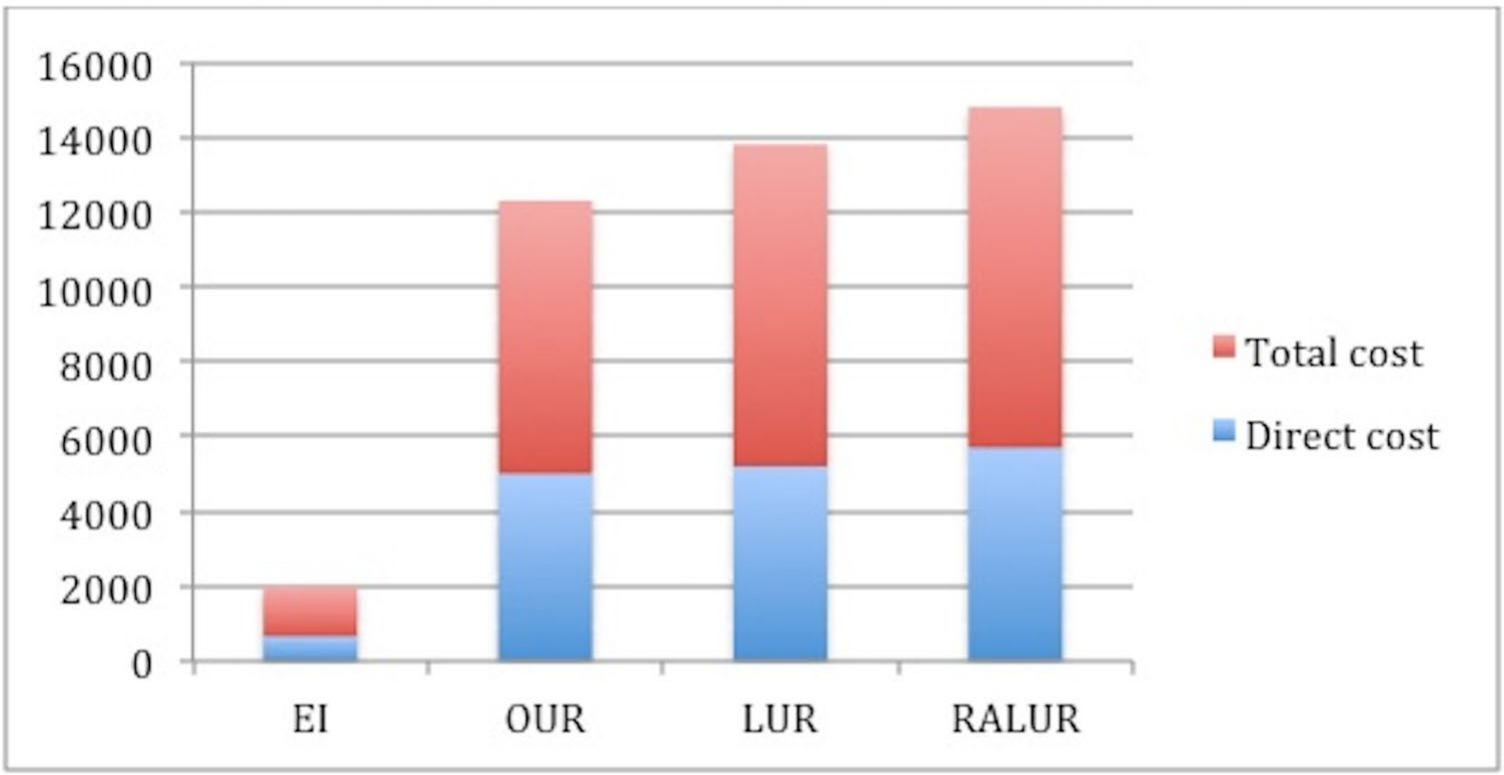
Comparison of direct and total costs between open ureteral reimplantation and robot-assisted laparoscopic ureteral reimplantation
3. Radiation exposition
  All patients underwent a pre-operative VUCG. 100% of patients in group A underwent a second intra-operative control VUCG. For each patient who underwent 2 EI, 4 VUCG were performed, for those who underwent 3 EI or 2 EI and surgery, 6, while for those who needed surgery after 3 EI, 8 with a median of 2.78 VUCG for each patient in group A [2–8]. In group B, the median exposition rate due to the VUCG was 1.45 [1–3].
  The long-term outcomes (success rate, redo-surgery, costs and X-ray exposure) for groups A, B, B1, B2 and B3 are described in Table 6.

**Table 6:** Secondary outcome, comparison between group A (endoscopic injection), B1 (open ureteral reimplantation), B2 (laparoscopic ureteral reimplantation) and B3 (robotic ureteral reimplantation)

	EI (Group A) *n* = 232	UR (Group B) *n* = 168	OUR (Group B1) *n* = 92	LUR (Group B2) *n* = 41	RALUR (Group B3) *n* = 35	*P* value
Success rate, *n* (%)	155 (66.81)	18 (89.88)	(91.3)	(87.8)	(88.57)	*P* < 0.01
Redo surgery, *n* (%)	63 (27.15)	5 (2.97)	3 (3.26)	1 (2.41)	1 (2.85)	*P* < 0.01
Direct cost (euros)	659		5000	5200	5700	*P* < 0.01
Total cost (euros)	1331	8330	7300	8600	9100	*P* < 0.01
X-ray exposure	2.78	1.45	1.53	1.4	1.21	*P* = 0 .87

## Discussion

Based on the clinical presentation and the grade of VUR, different therapeutic options are recommended [4]: in most cases of low-grade VUR, spontaneous resolution or non-operative-management with CAP is sufficient [9]. Traditionally, in case of failure, the only available alternative was open surgery. Nowadays, open [10], laparoscopic [11] and robot-assisted ureteral reimplantation can be proposed as safe procedures with a success rate of about 98% [12]. In these last decades, endoscopic injection was more and more used up to become the gold standard therapy for VUR of grades I and II [5, 13]. Despite all these therapeutic options, there are no guidelines about the best approach for intermediate and moderate III, IV and V grade of VUR [6]. No studies have been proposed yet comparing the outcome of endoscopic injection and ureteral reimplantation (open, laparoscopic and robotic) on treating III, IV and V grade of VUR.

We present the first international sample comparing endoscopic injection, open, laparoscopic and robot-assisted laparoscopic ureteral reimplantation in the pediatric population.

As reported in literature, no major intra-operative complications were registered in all groups [14–16], but there were post-operative complications with a rate of 5.25%. Specifically, post-operative complication rate was 4.31% after EI and 9.52% after UR without any significant differences neither between A and B group or among B1, B2 and B3 (*p* < 0.05). Literature data about complication rate in VUR treatment are extremely variable. In the previous comparative study written by Elsayed et al., post-operative complication rates between endoscopic group and laparoscopic treatment have been already compared: in the endoscopic group, postoperative complications were found in 13.3% of patients, while in the other one, 26.6% had complications with a statistically significant difference (*P* = 0.003) [16]. In Kurtz et al.’s study which analyzed open and robotic reimplantation, there were not post-operative complications [14], while complication rate after robotic reimplantation was 2.7% in Silay et al. [17], 10% in Grimsby et al. and Akhavan et al. [18, 19] and 30% in Marchini et al. [20]. This wide variability in the complication rate reported in literature may be due to different experiences among different centers and to reporting bias.

EI is not only associated with a lower complication rate, but it has also different advantaged such as a lower mean operative time, a better post-operative pain control, a reduced use of antibiotics and a lower length of hospital stay if compared to the ureteral reimplantation. These results are in accordance with those reported in literature. In our series, operative time was 28.9 min for endoscopic injection vs 28.6 in Elsayed et al. [16] and 41.5 in Kenneth et al. [15], 115 min for open surgery vs 110 min in Elsayed et al. [16] and 180 min in Kurtz et al. [14]; 198.5 min for laparoscopy vs 147 in Kenneth et al. [15] and 240 for robotic ureteral reimplantation vs 232 min in Kurtz et al. [14] with a statistically significant difference between the two groups (*P* < 0.001).

Furthermore, length of hospital stay after the endoscopic treatment was shorter than that after the surgical approach exactly as confirmed by literature (1.03 days for EI vs 1 day in Elsayed [16], 8.14 days after open surgery vs 2 [1–3] in Kurtz et al. [Kurtz], 6.81 days after laparoscopy vs 4 days [2–7] in Elsayed et al. [16] and 7.07 after robotic surgery vs 2 days [1–2] in Kurtz et al. [14] with a statistically significant difference (*P* < 0.001).

As we see in literature, minimally invasive surgery needs a 12% longer operative time [21], but it allows a better post-operative pain control, a reduced use of antibiotics, a lower length of hospital stay and a better cosmetic results in terms of position and length of cutaneous scars than the open traditional one [22, 23].

If we compare the secondary outcomes of our series with previous data reported in Literature, we get different results. In our series, efficacy of EI considered as absence of recurrences was 66.81% vs 73% in Tessier et al. [24] and 80% in Elsayed et al. [16]. It depends on the grade of reflux: SR decreases as the grade of VUR increases. These data are very similar to those reported in literature: success of subureteric injection was 71% of grade III as shown in the meta-analysis reported by Roth et al. [25], a little bit higher in a large systematic review published by Elder et al. [26].

SR of ureteral reimplantation is 89.88% which is significantly higher than that of EI, in accordance with literature data especially if we focus on the moderate and the high grade of VUR. These results are variable depending on the type of the technique used. Thanks to its low recurrence and complications rates, open surgery has been considered as the gold standard procedure for VUR treatment for long time [27, 28]. SR of open surgery was 91.3% in our series vs 93.5% in Tessier et al. [24] without any statistically significant difference (*p* < 0.05). In our cohort reflux resolved in 87.8% cases vs 93.75% reported in Elsayed et al. [16] and 96% in Riquelme et al. [11] and Esposito et al. [6], but in 77.2% in Tessier et al. [24] after the laparoscopic ureteral replantation with very discordant results. Finally, results in terms of SR after robotic ureteral reimplantation are extremely variable too: 88.57% in our cohort vs 97.9% in Silay et al. [17] and 99.3% in Kurtz et al.) [14], but 77% in Grimbsy et al. [18]. The wide variability of these results especially after minimally invasive surgery could be explained by the experience in minimally invasive surgery of the center, the role of surgeons who performed the procedures, the level of the center and the technical competences of the board which may increase the risk of complications, recurrences and failures.

Rate of redo-surgery after endoscopic injection was comparable with that reported in Literature (27.15% vs 23.7% in Tessier et al.) [24] and it was more frequent than that after ureteral reimplantation (27.15% vs 2.97% *p* < 0.00001) in accordance with the results of other studies such as Tessier’s which compared the RRS after EI and after laparoscopy (*p* = 0.02) [24]. If we compare the RRS among the three groups of UR, we can see that redo-surgery was more frequent after OUR (3.26% of total B1, 37.5% of failed B1) than after RALUR (2.85% of total B3, 25% of failed B3) and laparoscopic Lich Gregoir one (2.41% of total B2, 20% of failed B2) even if without a statistically significant difference (*p* = 0.87). Different results were reported in in Tessier et al. in which redo-surgery was more frequent after laparoscopic Lich Gregoir than after open surgery (5.7% vs 0%, p < 0.001) [24].

The cost effectiveness has been already considered as an important parameter in decision making of VUR treatment. Different results have been found about the intraoperative costs of EI and UR: while in our cohort and in Garcia-Aparicio et al., the direct cost of EI is significantly lower than that of UR [29]; according Kobelt et al. and Elsayed et al., there was no statistically significant difference between the median cost of one injection and the UR [16, 30]. All these studies made a comparison only between EI and OPEN UR. Our analysis and literature agree that the overall costs are known to be higher for ureteral reimplantation than for endoscopy [6, 29, 30].

Furthermore, we found that two or even three EI still cost less than one open, laparoscopic or robotic UR, Garcia-Aparicio et al. wrote that the significant variance in cost-effectiveness after one injection and after one reimplantation disappears after two injections [29].

Costs are higher in the robotic group than in open and laparoscopic ones, but there is no a statistically significant difference. This is probably due to a mitigation of costs obtained thanks to the use of robot in those non-pediatric centers with a concentration of activities and surgeons with more exposure to procedures may need a robotic approach [31, 32].

We analyzed the influence of age at surgery on the outcome of all the different therapeutic approach and we found out that complication and re-do surgery rates increased with the increasing of age at surgery for EI, but they decreased in B group (the tendency to decrease is higher in B3 than in B2 than in B1). The success rate had the opposite trend: it decreased with the increasing age at surgery in EI group, but it increased in in the other groups. No other studies have previously described the role played by the age of patients at treatment of VUR.

About the long-term effects of X-ray exposition in children, it has been demonstrated that 25% of radiation exposure to pediatric population is provided during urological evaluation [33]. Specifically during VUCG performed to assess the presence and the grade of VUR, gonads receive an unsignificant dose of radiation with a consequent increasing risk of gonadal tumors, leukemia, lymphomas and genetic deaths [34]. As reported in previous studies, there are alternative solutions to reduce the use of VUCG in this group of patients. First of all, the use of strict parameters can reduce the radiation dose absorbed at the skin entrance and the uniform whole body effective dose: the X-ray source should be set at a low dose mode, collimating to the smallest area possible [35]. Furthermore, immediate postoperative VUR assessment and long-term monitoring of patients with VCUG after EI or UR has been demonstrated to be unnecessary, due to the high cure rates with both treatment options. Follow-up VCUGs should be triggered by the occurrence of symptomatic UTIs than by recurrent or persistent VUR at VUCG [36].

This is the case of our protocol which proposed multiples postoperative control VUCG.

VUCG follow-up should be planned based on VUR grade: Thompson et al. proposed to delay the schedule of VCUG from yearly to every 2 years in children with mild VUR and every 3 years in children with moderate/severe VUR yields; following this pattern substantial reductions in the average numbers of VCUGs means a subsequent decrease in X-ray exposure [37]. Finally, newer non-ionizing technologies such as colour-flow Doppler ultrasonography (DUS) and started to be proposed as safe and efficient alternatives to VCUG in the diagnosis, screening and following of untreated or recurrent VUR avoiding the danger of exposure to ionizing radiation and the unpleasant catheterization in children [38, 39].

## Conclusion

In our study, the radiation exposure, the risk of recurrences and redo-surgery is significantly higher after endoscopic treatment than after ureteral reimplantation, especially for high grade of reflux (IV and V). Despite all these disadvantages, there is a statistically significant difference between EI and UR in terms of complication rate, LOS and overall costs.

Comparing OUR, LUR and RALUR, there is no significantly difference in terms of rate of complications and LOS as well as success rate and redo-surgery, but minimally invasive surgery has higher direct costs than open surgery, better cosmetic results and better post-operative pain control. To conclude, for grade III of VUR, EI is an excellent option both in terms of cost and success rate, but for higher grades, the therapeutic strategy has to be carefully chosen considering all the analyzed parameters. RALUR should be implemented with caution, particularly at sites with limited pelvic robotic experience and in non-pediatric centers where other specialties may offset the robotic related costs. Future prospective studies will determine if and whether minimally invasive techniques are justified as first-line treatment for high-grade reflux.

## Limits of the study

Even if selection criteria of patients have been strictly defined and patients were followed up for at least 5 years, some limits must be acknowledged. First of all, this is a retrospective study including two different groups of patients managed in three different institutions.

In one of these three centers, systematic voiding cystorurethrograms is still performed even if it does not change the management of asymptomatic patients. Finally, comparisons between the four groups of patients in terms of VUR grade, complications, success rate, costs and radiation exposure were not performed with a multivariable model adjusting for age, gender, presence of comorbidity, and hospital.

## Data Availability

No data sets were generated or analysed during the current study.

## Abbreviations

VUR: Vesicoureteral reflux
UTI: Urinary tract infection
CAP: Continuous antibiotic prophylaxis
EI: Endoscopic injection
UR: Ureteral reimplantation
OUR: Open ureteral reimplantation
LUR: Laparoscopic ureteral reimplantation
RALUR: Robot-assisted laparoscopic ureteral reimplantation
PUV: Posterior urethral valve
POM: Primary obstructive megaureter
VUCG: Voiding cystourethrogram
US: Ultrasound
Uro-MRI: Uro-magnetic resonance imaging
CRP: C-reactive protein
DMSA renal scan: Dymercaptosuccinil acid
PNF: Pyelonephritis
UHN: Uretero-hydronephrosis
APD: Antero-posterior diameter
VCS: Vescical catheter stay
DS: Drainage stay
LOS: Length of hospital stay
SR: Success rate
RRS: Rate of redo-surgery
